# The reasons for betel-quid chewing scale: assessment of factor structure, reliability, and validity

**DOI:** 10.1186/1472-6831-14-62

**Published:** 2014-06-03

**Authors:** Melissa A Little, Pallav Pokhrel, Kelle L Murphy, Crissy T Kawamoto, Gil S Suguitan, Thaddeus A Herzog

**Affiliations:** 1 University of Hawaii Cancer Center, 701 Ilalo St., B4, Honolulu, HI 96813, USA; 2 University of Hawaii Department of Kinesiology and Rehabilitation Science, 1337 Lower Campus Road PE/A 231, Honolulu, HI 96822, USA; 3 University of Guam Cancer Research Center, UOG Station, Mangilao, GU 96923, USA

**Keywords:** Areca nut, Betel-quid, Reasons for chewing, Betel nut

## Abstract

**Background:**

Despite the fact that betel-quid is one of the most commonly used psychoactive substances worldwide and a major risk-factor for head-and-neck cancer incidence and mortality globally, currently no standardized instrument is available to assess the reasons why individuals chew betel-quid. A measure to assess reasons for chewing betel-quid could help researchers and clinicians develop prevention and treatment strategies. In the current study, we sought to develop and evaluate a self-report instrument for assessing the reasons for chewing betel quid which contributes toward the goal of developing effective interventions to reduce betel quid chewing in vulnerable populations.

**Methods:**

The current study assessed the factor structure, reliability and convergent validity of the Reasons for Betel-quid Chewing Scale (RBCS), a newly developed 10 item measure adapted from several existing “reasons for smoking” scales. The measure was administered to 351 adult betel-quid chewers in Guam.

**Results:**

Confirmatory factor analysis of this measure revealed a three factor structure: reinforcement, social/cultural, and stimulation. Further tests revealed strong support for the internal consistency and convergent validity of this three factor measure.

**Conclusion:**

The goal of designing an intervention to reduce betel-quid chewing necessitates an understanding of why chewers chew; the current study makes considerable contributions towards that objective.

## Background

Betel-quid is chewed by approximately 600 million people globally [1], mainly in South Asia, Southeast Asia, and the Pacific islands, and is the fourth most commonly consumed psychoactive substance worldwide behind alcohol, nicotine, and caffeine [2,3]. It is reported to influence both the sympathetic and parasympathetic nervous systems causing both stimulatory and relaxing effects [4]. In many countries, betel-quid chewing is a socially accepted habit that is integrated into both ceremonial situations and routine aspects of daily life [5]. Betel-quid is most commonly the combination of areca nut (the seed of the palmaceous Areca catechu tree), piper betel leaf (a common vine), slaked lime (calcium hydroxide), and tobacco, though the ingredients vary by region, country, ethnicity, and personal preference [4,6]. In Guam, the areca nut often is referred to colloquially as “betel nut,” although this term can also refer to the areca nut chewed alone or as a betel-quid containing piper betel leaf, slaked lime, tobacco, and other ingredients such as spices (e.g., clove). In the current study, the term “betel-quid” refers to any preparation of chewed areca nut, including the nut alone and all admixtures involving betel leaf, slaked lime, tobacco, and other ingredients.

Given the number of health issues associated with chewing betel-quid, particularly oral cancer and precancerous conditions such as leukoplakia and oral submucous fibrosis [7], understanding ways to reduce betel-quid chewing is of global public health importance. In the last decade, betel-quid has been classified as a Group 1 carcinogen by the International Agency for Research on Cancer [6,8]. However, to date, most research on betel-quid chewing has been limited to epidemiological and biological investigations [9,10]. Limited research has been conducted to understand the behavioral and psychosocial factors that lead individuals to initiate and/or maintain betel-quid chewing. Determining such psychosocial and behavioral risk factors would help design prevention and treatment programs aiming to reduce the prevalence of betel-quid chewing.

Social learning theories [11,12] posit that substance use behaviors are motivated by expectancy reasons: that is, individuals use a substance because of the positive outcomes associated with the use. For example, individuals may continue to use an addictive substance because they like the way the substance makes them feel. Thus there may be several social, cultural, behavioral, and physiological reasons that influence substance use initiation and maintenance [13]. From prevention and treatment point of views, it is important to understand such reasons so that strategies may be developed to help individuals overcome an addiction.

Thus far, the reasons for betel nut chewing are poorly understood. The only existing study in the area is a study conducted among Taiwanese taxi cab drivers [14], which suggests that individuals chew betel-quid for some of the same reasons that individuals smoke tobacco [15]: to alleviate boredom, facilitate socialization, and promote activity and concentration at work. Currently, there is no standardized instrument to assess the variations in reasons for betel-quid chewing in populations of chewers. Such an instrument would help determine the main motivational factors influencing betel-quid dependence. In this study, we tested whether self-report items previously validated to assess reasons for tobacco smoking could be adapted to assess reasons for chewing betel-quid. Kuo and Lew-Ting’s (2008) findings suggest that reasons for smoking scales could be adapted to measure reasons for betel nut use. It should be noted that betel-nut’s psychoactive properties are similar to those of tobacco [14].

Specifically, we tested pertinent items from three measures well-studied in the tobacco use literature: the Modified Reasons for Smoking Scale [16], the Smoking Consequences Questionnaire [15], and the Wisconsin Inventory of Smoking Dependence Motives [17]. Items from these instruments were modified from “reasons for smoking” to evaluate reasons for chewing betel-quid and were expected to represent constructs such as reinforcement (positive and negative), social/cultural, and stimulation. Items covering factors that may be unique to the betel-quid chewing culture, such as whether it is considered rude not to chew in social situations (e.g., when participating in ceremonies) or whether chewing is associated with social status also were added to the measure. In the current study, we aimed to validate the reasons for chewing scale among a sample of English-speaking male and female betel-quid chewers living in Guam using confirmatory factor analysis. We tested whether the factor structure previously established for reasons for smoking among smokers replicated for reasons for betel-quid chewing among betel-quid users. Further, we tested whether the confirmatory model was invariant across gender. We also assessed the scales psychometric properties. We predicted that the measure would demonstrate good internal consistency, measured through Cronbach's alpha, and convergent validity, assessed using analysis of variance (ANOVA) and multiple linear regression analyses.

## Methods

### Procedure

Data reported in the current analyses were obtained as part of a larger study that examines psychological, behavioral, and cultural issues related to areca nut and betel-quid chewing among self-identified betel-quid chewers and ex-chewers in Guam. Participants were recruited through newspaper advertisements, flyers, and community events in Guam. At the initial screening, potential participants were asked a series of questions to determine chewer status. For the current analyses, participants (*N* = 351) were limited to self-identified chewers who reported chewing for at least three years, and at a current rate of at least once per week. Informed written consent was obtained prior to participation in the study. Surveys were administered in person at community events (98%) and through the mail (2%). Participants who completed the survey in the mail were given a stamped and addressed return envelope. Following receipt of completed questionnaires, all participants were provided with a $25 gift card. The research protocol for this study was approved by the University of Guam Institutional Review Board and the University of Hawaii at Manoa Institutional Review Board.

### Participants

Participants consisted of 351 adult current betel-quid chewers in Guam. Half the sample was male (50.1%), and 59.3% had completed high school. The average age was 35.6 years old (SD = 20.9). The ethnic distribution of the sample was as follows: 34.5% Chamorro, 27.9% Chuukese, 21.7% Palauan, 6.6% Yapese, and 9.3% other (e.g., Carolinian, Filipino, Marshallese). Participants reported chewing for a mean of 15.3 years (SD = 12.8) and 11.9 times per day (SD = 13.7). Three-quarters of participants (75.3%) reported chewing every day. The majority of participants (66.1%) reported adding tobacco to their betel-quid, compared to only 13.7% who reported chewing areca nut alone (i.e., as the sole ingredient of the betel-quid).

### Measures

#### Reasons for betel-quid chewing scale (RBCS)

The RBCS is a 10-item scale to measure the motives for chewing betel-quid, adapted from existing scales assessing reasons for smoking [15-17] (see Table 1 for a list of specific items and hypothesized constructs). Responses were made on a five-point scale (0 = Not important to 4 = Extremely important). The three chewing motive scores were hypothesized and computed: reinforcement, social/cultural, and stimulation.

**Table 1:** Mean (SD) of individual items in the reasons for betel-quid chewing scale (RBCS)

**RBCS items**	**Mean**	**Standard deviation**	**r**	**Factor loadings**
*Reinforcement construct*	1.78	1.40		
1. I like the taste	1.95	1.51	.92	.75
2. I like to have something in my mouth at all times	1.58	1.52	.92	.94
*Social/cultural construct*	1.26	1.08		
3. All of my friends chew	1.63	1.42	.81	.78
4. My family members chew	1.74	1.37	.78	.73
5. It’s rude not to chew	0.88	1.35	.83	.63
6. People will not respect me if I don’t chew	0.73	1.27	.81	.62
*Stimulation construct*	2.24	1.30		
7. It relaxes me	2.54	1.41	.84	.66
8. It gives me energy	2.51	1.52	.88	.74
9. It helps me make decisions	1.68	1.64	.81	.79
10. I like the way it makes me feel	2.18	1.54	.86	.85

r = corrected item-total correlation. Factor loadings are standardized. Response options ranged from 0-4.

#### Demographics

Information related to age, gender, education, and ethnicity was collected. Ethnicity was assessed by an open-ended item asking participants to indicate “the one ethnic group you most identify with.”

#### Betel-quid consumption and dependence

Betel-quid consumption was assessed through three items: number of chews per day, number of years as a chewer, and the type of betel-quid chewed (i.e., areca nut alone, betel-quid without tobacco, or betel-quid with tobacco). There is evidence suggesting a dependency syndrome related to betel-quid use [18]. Therefore, we chose to assess dependency using the 16-item Betel-quid Dependence Scale (BQDS) [19,20]. Each item in the BQDS employs a dichotomous outcome (0 = No to 1 = Yes). The BQDS score was calculated by summing the 16 items and then dividing by the total number of items so that each score represented the proportion of items endorsed.

#### Other items related to reasons for chewing

Participants were asked if they began chewing because they liked the taste (0 = No or 1 = Yes). Chewing among family members was assessed by asking participants: “What other members of your family chew betel nut?” Response options, which included children, brothers, sisters, parents, grandparents, aunts, uncles, and other family members (0 = No to 1 = Yes), were summed to create a chewing among family members score (range 0-8). To assess social/cultural reasons for chewing, participants were also asked, “Is not chewing betel nut in social situations considered an insult?” (0 = Never to 4 = Almost always). Finally, participants were asked to rate on a 5-point scale how important chewing betel-quid was in the following situations: birthdays, fiestas, anniversaries (of deaths), parties, rosaries, and working (6 items; 0 = Not important to 4 = Extremely important; α = 0.93).

### Data analysis

To assess the hypothesized factor structure of the RBCS, a confirmatory factor analysis was performed using maximum likelihood estimation, with Mplus version 7 [21]. This model included reinforcement as a latent variable with two items as indicators, social/cultural as a latent variable with four items as indicators, and stimulation as a latent variable with four items as indicators. Full information maximum likelihood estimation was used for estimating missing data and MLR estimation for non-normally distributed data, with Mplus version 7 [21]. To ensure adequate fit of the models, rigorous evaluation criteria were adopted. A chi-square test was chosen as the statistical test of model fit (α = .05). Because this test can be sensitive to minor deviations in model fit in large samples [22], the comparative fit index (CFI), Tucker-Lewis index (TLI), root mean square error of approximation (RMSEA), and standardized root mean square residual (SRMR) also were used to evaluate the model fit [23,24]. The following cut-offs were employed for establishing adequate fit: CFI ≥ .95 [25]; TLI ≥ .95 [25]; RMSEA < .05 [22]; SRMR < 1.0 [25]. Factor loadings were determined to be robust if they were > .40.

The measurement invariance of the hypothesized model across genders was tested by assessing the equivalence of the configural model and factor loadings across genders using nested-model chi-square difference tests within the multiple-group comparison framework. First, the configural model was tested in the overall sample. Next, the model was run separately for each gender to determine configural equivalence. Gender equivalence of factor loadings was then tested by comparing the nested model (i.e., the model with factor loadings constrained across genders) to the base model (i.e., the model with factor loadings freely estimated across genders) using the nested-model chi-square difference test. A significant chi-square difference would mean that constraining the factor loadings significantly deteriorated the fit of the base model and would thus indicate one or more factor loadings were significantly variant across genders [26].

We examined the internal consistency of the overall scale and the corresponding subscales using Cronbach's alpha. Convergent validity of the RBCS was assessed using analysis of variance (ANOVA) and multiple linear regression analyses in SAS 9.3 [27]. ANOVA was conducted with Least Square Mean (LSM) comparisons to assess mean differences across groups as a function of each of the RBCS subscales (reinforcement, social/cultural, stimulation). Groups included betel-quid chews per day (<5, 5-9, 10-15, ≥16), quid type (nut alone, quid (lime, leaf, nut), and quid plus tobacco), ethnicity (Chamorro, Chuukese, Palauan, Yapese, other), and age (18-25, 26-35, 36-45, >45). Multiple comparisons across means were conducted using Tukey’s Studentized Range (HSD) test. Multiple linear regressions were examined across several correlates of the three reasons for betel-quid use factors, namely: liking the taste of betel-quid as the reason for initiation, the beliefs that not chewing is a cultural insult and chewing is socially important, chewing among family members, chews per day, the BQDS and inclusion of tobacco in betel-quid. Models controlled for age, gender, education and ethnicity (Chamorro, Chuukese, Palauan, and Yapese versus other) and separate models were run with each of the three RBCS constructs specified as dependent variables. Statistical tests were two-tailed, with significance was set at p < .05.

## Results

### Confirmatory factor analysis (CFA)

The hypothesized model fit the data adequately (see Table 2). Items loaded significantly on their respective factors. Modification indices called for the error covariance between items 5 and 6, and items 7 and 8 to be freely estimated. After making these model modifications, the model fit the data better: χ^2^ = 153.62, df = 30, p < .0001; RMSEA = 0.11, 90% CI = 0.09, 0.13; CFI = 0.93; TLI = 0.90; SRMR = 0.06. The multi-group comparison of the model across gender suggested that the model was invariant across gender. The chi-square difference test indicated that the fit of the model when the factor loadings were constrained to be equal across gender was not significantly different from the fit of the model when factor loadings were estimated freely across gender (Chi-square difference [10] = 11.18, *p* = .34; see Table 2, Models 2 and 2a).

**Table 2:** **Fit indices for confirmatory factor analyses (*N*** **= 351; *n***_**males**_ **= 176; *n***_***females***_ **= 175)**

**Model**	**χ**^**2 **^**(d.f)**	**CFI**	**TLI**	**RMSEA (90% CI)**	**SRMR**	**χ**^**2 **^**difference test**
*Full sample*						
1 Hypothesized measurement model	340.67*(32)	.84	.77	.17 (.15, .18)	.10	
1a Modified measurement model	153.62*(30)	.93	.90	.11 (.09, .13)	.06	
*Gender multi-group analysis*						
2 Full model	196.31* (67)	.93	.91	.11 (.09, .12)	.07	∆ χ^2^ = 11.18, d.f. = 10, p = .34
2a Nested model	207.49* (77)	.93	.92	.10 (.08, .12)	.07	

*Note.* CFI: comparative fit index; TLI: Tucker-Lewis index RMSEA: root mean-square error of approximation; and SRMR: root mean square residual.

*p < .05.

### Reliability and convergent validity

The total RBCS score, as well as the three subscales identified in the CFA model, had good internal consistency. The Cronbach’s alpha for the overall 10-item RBCS was .88. Similarly, the alpha for the subscales pleasure and social/cultural was .82, and stimulation was .87. The means, standard deviations, item-total correlations, and factor loadings are shown in Table 1.

Table 3 displays the means for the RBCS factors by chews per day, quid type, age, and ethnicity. Significant differences across chews per day were found on reinforcement (F_3, 340_ = 11.70, p < .0001), social/cultural (F_3,__338_ = 9.41, p < .0001) and stimulation scales (F_3, 340_ = 6.10, p = 0.001). Chewers that reported ≥16 chews per day endorsed significantly higher levels on the subscales reinforcement and social/cultural subscales compared to all other groups and higher levels on stimulation compared to chewers reporting <5 and 10-15 chews per day. Chewers reporting 10-15 chews per day endorsed significantly higher levels on the reinforcement and social/cultural subscales as reasons to chew compared to chewers reporting <5 chews per day. There were no differences observed between chewers reporting <5 and 5-9 chews per day. Significant differences across quid type were found on reinforcement (F_2, 340_ = 3.52, p = 0.03), social/cultural (F_2, 338_ = 4.28, p = 0.01) and stimulation subscales (F_2, 340_ = 12.54, p < .0001). Quid plus tobacco chewers had significantly less endorsement on the subscale reinforcement compared to quid alone. Quid chewers had significantly higher levels of endorsement on the social/cultural subscale compared to those who chewed nut alone. Nut alone chewers had significantly less endorsement on the stimulation subscale compared to the other groups. Significant differences across age groups were found on reinforcement (F_3, 340_ = 5.26, p = 0.002) and stimulation scales (F_3, 340_ = 3.54, p = 0.01), but not the social/cultural scale (F_3,__338_ = 0.78, p = 0.51).Young adults (18-25) had significantly lower levels of endorsement on the reinforcement subscale compared to those over age 35. Individuals in the 26-35 year old age group reported higher endorsement on the stimulation subscale compared to those over age 35. Significant differences across ethnic groups were found on reinforcement (F_4, 339_ = 16.22, p < .0001) and stimulation scales (F_4, 339_ = 17.62, p < .0001), but not the social/cultural scale (F_4, 337_ = 1.00, p = 0.41). Chuukese had significantly lower levels of endorsement on the reinforcement subscale, but significantly higher levels of endorsement on the stimulation subscale compared to the other ethnic groups.

**Table 3:** RBCS factors as a function of chews per day and quid type

	**Mean CPD**	**Reinforcement**	**Social/cultural**	**Stimulation**
*Chews per day (CPD)*				
<5 (n = 78)	2.78 (0.88)	1.32 (1.13)^a^	0.85 (0.78)^a^	1.89 (1.41)^a^
5-9 (n = 87)	6.49 (1.36)	1.44 (1.41)^ab^	1.17 (0.86)^ab^	2.34 (1.22)^ab^
10-15 (n = 109)	11.59 (2.11)	1.88 (1.45)^b^	1.28 (1.15)^b^	2.08 (1.32)^a^
≥16 (n = 77)	27.62 (21.91)	2.47 (1.27)^c^	1.73 (1.27)^c^	2.70 (1.06)^b^
*Quid type*				
Nut alone (n = 50)	6.94 (6.54)	1.99 (1.26)^ab^	0.93 (0.99)^a^	1.42 (1.13)^a^
Quid (lime, leaf, nut) (n = 69)	9.39 (7.15)	2.09 (1.43)^a^	1.56 (1.33)^b^	2.16 (1.38)^b^
Quid plus tobacco (n = 232)	13.68 (1.24)	1.63 (1.41)^b^	1.23 (1.00)^ab^	2.43 (1.24)^b^
*Age*				
18-25 (n = 121)	9.20 (6.02)	1.40 (1.37)^a^	1.24 (0.88)^a^	2.34 (1.29)^ab^
26-35 (n = 83)	14.47 (23.68)	1.83 (1.38)^ab^	1.40 (1.06)^a^	2.53 (1.12)^a^
36-45 (n = 68)	14.09 (9.86)	2.00 (1.37)^b^	1.36 (1.21)^a^	1.97 (1.32)^b^
>45 (n = 79)	11.39 (9.56)	2.12 (1.39)^b^	1.54 (1.23)^a^	2.00 (1.42)^b^
*Ethnicity*				
Chamorro (n = 121)	9.67 (7.14)	2.21 (1.33)^ab^	1.38 (1.25)^a^	1.94 (1.29)^a^
Chuukese (n = 98)	8.80 (6.90)	0.97 (1.17)^c^	1.27 (0.84)^a^	3.08 (1.03)^b^
Palauan (n = 76)	14.43 (9.02)	1.71 (1.43)^a^	1.13 (1.08)^a^	1.83 (1.20)^a^
Yapese (n = 23)	29.78 (41.66)	2.72 (0.93)^b^	1.00 (1.10)^a^	2.23 (1.12)^a^
Other (n = 33)	10.85 (6.35)	2.08 (1.36)^ab^	1.24 (1.10)^a^	1.76 (1.29)^a^

mean (sd) are reported in the table.

^a,b,c,^: For each of the three variables, the same subscript letters after the mean (se) numbers for the level designate that the levels were statistically insignificant (p > 0.05), different letters indicate significant differences across group comparisons (p < 0.05).

Results of the multiple linear regressions are shown in Table 4. The reinforcement construct was significantly associated with the following variables: initially chewing because the taste was enjoyable, believing that not chewing is a cultural insult, believing that chewing is socially important, and having higher levels of betel-quid dependence. The construct social/cultural was significantly associated with: believing that not chewing is a cultural insult, having family members who chew, and believing that chewing is socially important. The stimulation construct was significantly associated with believing that chewing is socially important and having higher levels of betel-quid dependence.

**Table 4:** Summary of the linear regression analyses testing correlates of the RBCS

	**Reinforcement**	**Social/cultural**	**Stimulation**
Began chewing because enjoyed the taste	0.20 (0.05)**	−0.04 (0.05)	−0.03 (0.05)
Not chewing is a cultural insult	0.09 (0.04)*	0.17 (0.05)*	−0.03 (0.05)
Family members chew	0.00 (0.05)	0.11 (0.05)*	0.07 (0.05)
Chewing is socially important	0.41 (0.05)**	0.47 (0.05)**	0.36 (0.05)**
Chews per day	0.05 (0.05)	0.02 (0.05)	0.06 (0.05)
Betel-quid dependence	0.10 (0.05)*	0.10 (0.06)	0.28 (0.05)**
Chewing betel-quid plus tobacco	−0.10 (0.05)	−0.07 (0.06)	−0.01 (0.05)

Beta (se) are reported in the table. Models controlled for age, gender, education and ethnicity.

Variables standardized (mean = 0, std = 1).

**p < .0001; *p < .05.

## Discussion

Despite that betel-quid is one of the most commonly used psychoactive substances worldwide and a major risk-factor for head-and-neck cancer incidence and mortality globally, currently no standardized instrument is available to assess the reasons why individuals chew betel-quid. A measure to assess reasons for chewing betel-quid could help researchers and clinicians develop prevention and treatment strategies. In the current study, we sought to develop and evaluate a self-report instrument for assessing the reasons for chewing betel-quid. Thus, the current study is significant because it contributes toward the goal of developing effective interventions to reduce betel-quid chewing in vulnerable populations.

Specifically, a 10-item RBCS scale was created. The scale drew from several existing reasons for smoking scales [15-17]. We hypothesized that the 10-item RBCS included three subscales representing three constructs: reinforcement, social/cultural and stimulation. Results of the confirmatory factor analysis supported the three-factor model. Further analyses revealed that the three-factor model was equivalent across genders and the full 10-item scale and subscale scores exhibited good internal consistency.

The subscales appeared to correctly measure the constructs that they were hypothesized to measure. The construct validity of the subscales was partly demonstrated by the convergence between the scores on reasons for quitting subscales and chews per day: that is, self-reported chews per day tended to increase with higher endorsement of each of the three types of reasons for chewing; namely reinforcement, social/cultural, and stimulation. Moreover, regression analyses showed that the three subscales were significantly correlated with variables that were expected to correlate with them. For example, initiating chewing because of liking the taste of betel-quid was associated with higher scores on reinforcement as a reason for current chewing. Similarly, perception that betel-quid chewing was socially important was associated with higher scores on social/cultural reasons for current chewing, and betel-quid dependence was associated with higher scores on stimulation as a current reason for chewing.

We found that among the chewers of different quid types, chewers that added tobacco to their quid reported higher levels of stimulation compared to chewers of the nut alone. Among the three RBCS constructs, stimulation was the most strongly endorsed, followed by reinforcement, and social/cultural reasons. These results suggest that people are more likely to chew betel-quid because of the way it makes them feel. This is not surprising given that two thirds of the sample add tobacco to their quid, which have been demonstrated to increase use and dependence [20]. These results suggest that addressing the stimulating effects of chewing will be a key to designing interventions to help chewers quit. For example, the cognitive-behavioral therapy (CBT) approaches that have been found successful in smoking cessation [28] may be adapted for betel-quid cessation. In addition, pharmacotherapies may be developed to help betel-quid chewers quit chewing.

Ethnic group comparisons demonstrated that Chuukese reported the highest levels of stimulation compared to the other ethnic groups. Interestingly, there were no ethnic differences observed among the social/cultural reason subscale. However, believing that chewing is socially important was significantly related to all three constructs in the regression analysis. In many countries within the Western Pacific region, the long-established behavior of betel-quid use is integral to community life, from routine aspects of daily life to ceremonial celebrations [5]. Given the social importance of chewing betel-quid, chewers might fear the negative social repercussions associated with quitting. This idea is supported by the association between the RBCS constructs reinforcement and social/cultural and the belief that not chewing is a cultural insult. For instance, for an individual attending a social or cultural event where betel-quid is offered, refusing the betel-quid could be construed as an insult by the host. Thus interventions designed to treat or prevent betel-quid chewing may need to include a strong social/cultural component. For example, such interventions may provide chewers trying to quit chewing with skills regarding how to deal with the social/cultural pressures to chew. With regards to prevention, it is important to closely study the betel-quid chewing initiation process and what type of role social influence, including social norms and peer or family pressure, plays in initiation. If social influence is found to play an important role in chewing initiation among youth and young adults, perhaps social influence-based smoking cessation interventions that have been found to be effective among youth and young adults may be adapted to prevent betel-quid initiation.

The findings of the current study must be interpreted in light of several limitations. The sample was a small convenience sample from the Micronesian island of Guam. Although the sample included a broad range of chewers in terms of ethnicity, gender, and age, the results of the current study may not be representative of Guam chewers generally or chewers from other countries. Therefore, our results could over- or underestimate the associations seen in the general population of chewers. However, given that the RBCS performed in a fashion we expected and was associated with other measures of use, we believe that our sample does not pose a great threat to the validity of our results. Nonetheless, our results should be interpreted with caution.

Two-thirds of participants (66.1%) reported adding tobacco to their betel-quid. Because of the addictive properties of tobacco, it is possible that the effects of tobacco were confounded with the effects of areca nut. However, chewers that added tobacco to their quid did not differ significantly from chewers that did not add tobacco to their quid on their endorsement of social/cultural and stimulation subscales. Thus, we do not believe this was a significant threat to the validity of our study.

Additionally, since this study was conducted with established chewers who had reported chewing for at least three years, future studies should test this scale in a sample of newer users to see if this scale can elucidate reasons for initiation and early dependence of betel-quid. The RBCS also should be validated in a larger sample of chewers to assess the stability of this measure. Lastly, some people may chew betel-quid without an explicit reason. Therefore, it is possible that they would provide different answers to the same RBCS items at different points in time. Because we were using a cross sectional sample in the current study, we were not able to test predictive validity and test retest reliability of RBCS. Given that the RBCS could be a useful instrument for assessing determinants of betel-quid cessation, it is important to assess its predictive validity. Future research should explore these additional tests of validity and reliability in a longitudinal sample.

## Conclusions

Currently, there is a dearth of research exploring reasons for chewing betel-quid. However, in order to develop empirically supported treatments, researchers must first understand the reasons people engage in the behavior. The current study is novel in that it is the first study to develop and validate an assessment tool in an ethnically diverse sample of chewers that can be used to gain insight about the reasons people chew betel-quid. Researchers and practitioners can use the RBCS to develop culturally tailored betel-quid cessation and risk reduction programs. This is the first step in reducing the public health burden associated with betel-quid chewing worldwide.

### Role of funding source

Funding for this study was provided by National Cancer Institute (NCI) Grant U54 CA143727.

The NCI had no further role in study design; in the collection, analysis and interpretation of data; in the writing of the report; or in the decision to submit the paper for publication. M.A. Little was supported during the work on this project by a postdoctoral fellowship on NCI grant R25 CA90956.

## Competing interests

All authors declare that they have no conflict of interests.

## Authors’ contributions

ML and PP performed statistical analyses and wrote sections of the manuscript. TH and KM designed the study and wrote sections of the manuscript. CK and GS wrote the protocol and contributed to the survey design. All authors contributed to and have approved the final manuscript.

## Pre-publication history

The pre-publication history for this paper can be accessed here:

http://www.biomedcentral.com/1472-6831/14/62/prepub
